# Some Neglected Medical Obligations

**Published:** 1893-05-20

**Authors:** 


### The Hospital

AN INSTITUTIONAL JOURNAL OF

**Science, Medicine, Nursing, and Philanthropy.**

*For Hospitals, Asylums, Infirmaries, Dispensaries, Medical Practitioners, Students, Nurses, and the Charitable Public.*

VOL. XIV.—No. 347.] MAY 20, 1893.

### SOME NEGLECTED MEDICAL OBLIGATIONS.

EVERY medical man feels sometimes as if he must inevitably be swamped in his professional work amidst an overwhelming multiplicity of details and new theories. Not a few actually are swamped, and are all their lives conscious of tasks which they can by no possibility perform. It cannot be said that such a state of things is creditable to medicine; and it certainly would not be considered satisfactory by the people at large if they could by any means be made clearly to see it. The first duty of medical statesmanship at any given period is to provide a sufficient number of competent practitioners for the ordinary necessities of the people. It is necessary to insist upon this. Medical men who take a statesman's view of the duties and responsibilities of the profession are few, and tend to become fewer. The acutest medical intellects of the present time are devoted to two things, money-making and original research. This is gravely injurious, and diminishes the esteem in which medicine is held in exact proportion to the diminution of the services rendered to the public by the profession as a whole.

In order to be serviceable to the public in the highest degree, it is imperative that the average practitioner be thoroughly well taught and trained in the leading facts of bodily structure and function, and in those main principles of amelioration and cure which have been solidly established by experience and art. But this is exactly the opposite of what is generally done. Every teacher of anatomy tries to make every individual student a minute anatomist; every lecturer on the practice of medicine endeavours to make every student acquainted with every byeway, every hole and corner, every possible curiosity and exception which the human family has furnished to inquiring medicine from the earliest times; every examiner, in the true spirit of the pedant, goes more and more out of the beaten track in order that he may at least show the wide-ranging abundance of his own knowledge whether he bring out the knowledge possessed by the student or not. But there is no statemanship here; no comprehension of the true nature of public duty. There is childish vanity, the schoolboy's narrowness of view, and the schoolboy's inconsequence in action. But as for statesmanship, grasp of affairs, breadth of mind, and the suppression of the ego in response to the demands of great public occasions—these are nowhere to be found.

Take the average medical student or practictioner! Is it possible to make him a minute anatomist with the time and means at disposal? Is it possible to give him any intelligent comprehension of the thousand-and-one mysterious accumulations and exceptions of medical history? It is not possible! Though death were the penalty of failure, the thing could not be done. But it is possible to make the average student quite familiar with the leading facts of bodily structure and function, with the character and relations of the greater and more important organs of the body, with secretion, excretion, nerve action, and other great functions; with inflammation, blood changes, and other chief processes in disease. It is possible also to make him quite familiar with the leading principles of medical treatment; such, for example, as the putting of his patient under safe conditions, the subduing of inflammatory action, the relief of pain, the maintaining as far as possible of the chief natural functions of the body under adverse circumstances, the preserving of the strength until the forces of recovery have time to act, and so on. All this can be done, and done thoroughly. And this is what ought most religiously to be done, whatever else is left undone. The public trusts us to do these common things, and in this faith calls us to its help in emergencies of life and death. But these things, which are so insignificant in the eyes of pedantry, are the very soul and life of medical practice in the eyes of the practical medical statesman. They are so much, indeed, that they constitute all that the average medical student can accomplish, and all he ought to be expected to accomplish. Moreover, even these things he will not thoroughly accomplish without an infinity of repetition, the dealing with numerous actual examples and cases, and the most painstaking thoroughness and intelligence on the part of his teachers.

One of the most frequent complaints in the mouths of private patients is that of the want of thoroughness on the part of practitioners. The doctor runs in, they will tell you, looks at the tongue and feels the pulse, asks a civil question or two, says the patient is better, or soon will be, shakes hands, is gone, and has done—what? Nothing at all, think the patient and his friends: and too often this criticism is absolutely and entirely just. But why was the doctor so perfunctory and unsatisfactory? Because he had never been taught to be otherwise!

It is one of the commonest of daily experiences for a man of limited professional education to beat a more widely read physician hollow in actual practice. Indeed, some men of wide reading are complete failures at the bedside. Such persons soothe their disappointed self-love by dubbing patients undiscriminating fools, and successful doctors plausible charlatans. But that is mere nonsense. The real explanation most frequently is that the successful man has had the wit to master thoroughly those leading principles and details of diagnosis and treatment which are called for in every-day practice. This part of his business he has at his fingers' ends. But surely he is entirely right; he is only doing what plain sense and honest duty demand. In these pages we always contend for the utmost possible knowledge and the widest and deepest culture of all kinds. But there are certain things which must always take precedence in a practical man's affairs; and these are the common duties of his calling which differentiate him from every other man; the duties which the world looks for from him as naturally as it looks for warmth from the sun or rain from the clouds. The man who professes to the world that these are the duties he is prepared to do for it, but who never takes the trouble to learn how to perform them well, or who despises them because they are "common and merely practical," is nothing but a downright swindler. He may be in his own estimation a man of culture, in every way superior to the bustling practitioner round the corner. But in plain terms he is a make-believe and a fraud, and happily for them his non-professional neighbours have had the wit to find out the fact.

It cannot be too often insisted upon that all of us cannot be researchers; all of us cannot be medical philosophers. But we can all know the common principles of diagnosis and treatment, and the common daily necessities of our patients as well as the intelligent child knows his alphabet, or the bigger schoolboy his multiplication table. Let us know these whatever else we do not know; and let us keep open minds for the inflow of new truths as time and opportunity may offer.

The natural leaders of our profession do not grasp the reins of control and guidance with sufficient firmness. They acquiesce too timidly in the prevailing rage for universal original research. Every ambitious man feels that he must be a researcher, and every earnest clinician feels that he must be an apologist for his commoner work. But this is truly ridiculous. The researcher is still on his trial. His achievements, which are destined to endure, are as yet of scientific and academic value almost exclusively. For him to claim precedence over the accomplished clinician on the strength of his mere experimentations—resultless as they have too often been—is as if the acrobat who shows marvellous feats of swimming should demand more honour than the courageous diver who has saved a hundred lives from drowning. The leaders of medicine, who are responsible for its character and conduct, should leave nothing undone which may provide for the public a thoroughly-trained and disciplined army fitted for common medical service. The common work of our profession we must have done. The new, the extraordinary, and the marvellous we will also have done, if we can.

